# Nocturnal Blood Pressure Estimation from Sleep Plethysmography Using Machine Learning

**DOI:** 10.3390/s23187931

**Published:** 2023-09-16

**Authors:** Gizem Yilmaz, Xingyu Lyu, Ju Lynn Ong, Lieng Hsi Ling, Thomas Penzel, B. T. Thomas Yeo, Michael W. L. Chee

**Affiliations:** 1 Centre for Sleep and Cognition, Yong Loo Lin School of Medicine, National University of Singapore, Singapore 117549, Singapore; gizem.yilmaz@nus.edu.sg (G.Y.); lyux96@nus.edu.sg (X.L.); julynn.ong@nus.edu.sg (J.L.O.); 2 Centre for Translational Magnetic Resonance Research (TMR), Yong Loo Lin School of Medicine, National University of Singapore, Singapore 117549, Singapore; 3 Department of Cardiology, National University Heart Centre Singapore, Singapore 119074, Singapore; mdcllh@nus.edu.sg; 4 Department of Medicine, Yong Loo Lin School of Medicine, National University of Singapore, Singapore 117549, Singapore; 5 Interdisciplinary Center of Sleep Medicine, Charité—Universitätsmedizin Berlin, 10117 Berlin, Germany; thomas.penzel@charite.de; 6 Department of Electrical and Computer Engineering, National University of Singapore, Singapore 117549, Singapore; 7 N.1 Institute for Health and Institute for Digital Medicine (WisDM), National University of Singapore, Singapore 117549, Singapore; 8 Integrative Sciences and Engineering Programme (ISEP), National University of Singapore, Singapore 117549, Singapore; 9 Martinos Center for Biomedical Imaging, Massachusetts General Hospital, Charlestown, MA 02114, USA

**Keywords:** nocturnal blood pressure, photoplethysmography, cuffless blood pressure measurement, blood pressure estimation, cardiovascular health, sleep

## Abstract

Background: Elevated nocturnal blood pressure (BP) is a risk factor for cardiovascular disease (CVD) and mortality. Cuffless BP assessment aided by machine learning could be a desirable alternative to traditional cuff-based methods for monitoring BP during sleep. We describe a machine-learning-based algorithm for predicting nocturnal BP using single-channel fingertip plethysmography (PPG) in healthy adults. Methods: Sixty-eight healthy adults with no apparent sleep or CVD (53% male), with a median (IQR) age of 29 (23–46 years), underwent overnight polysomnography (PSG) with fingertip PPG and ambulatory blood pressure monitoring (ABPM). Features based on pulse morphology were extracted from the PPG waveforms. Random forest models were used to predict night-time systolic blood pressure (SBP) and diastolic blood pressure (DBP). Results: Our model achieved the highest out-of-sample performance with a window length of 7 s across window lengths explored (60 s, 30 s, 15 s, 7 s, and 3 s). The mean absolute error (MAE ± STD) was 5.72 ± 4.51 mmHg for SBP and 4.52 ± 3.60 mmHg for DBP. Similarly, the root mean square error (RMSE ± STD) was 6.47 ± 1.88 mmHg for SBP and 4.62 ± 1.17 mmHg for DBP. The mean correlation coefficient between measured and predicted values was 0.87 for SBP and 0.86 for DBP. Based on Shapley additive explanation (SHAP) values, the most important PPG waveform feature was the stiffness index, a marker that reflects the change in arterial stiffness. Conclusion: Our results highlight the potential of machine learning-based nocturnal BP prediction using single-channel fingertip PPG in healthy adults. The accuracy of the predictions demonstrated that our cuffless method was able to capture the dynamic and complex relationship between PPG waveform characteristics and BP during sleep, which may provide a scalable, convenient, economical, and non-invasive means to continuously monitor blood pressure.

## 1. Introduction

Hypertension is a leading risk factor for morbidity and mortality related to cardiovascular disease (CVD) [1]. Early diagnosis of hypertension is essential for the prevention and treatment of CVD and involves regular BP monitoring. Daytime blood pressure measurements taken intermittently may not effectively identify masked and nocturnal hypertension [2], which could provide a more accurate assessment of cardiovascular risk [3]. Specifically, night-time or nocturnal BP is more closely associated with cardiovascular or cerebrovascular events than daytime BP [4,5]. However, measuring BP during sleep with contemporary cuff-based systems can be uncomfortable and may interrupt sleep. For continuous, long-term use, cuffless BP estimation methods equipped with advanced machine learning (ML) algorithms have been proposed.

The feasibility of continuous cuffless BP measurement has recently been demonstrated using machine learning models trained on plethysmography (PPG) and electrocardiography (ECG) signals. ML can use PPG waveform features alone [6,7] or in combination with ECG features. The latter is used to calculate pulse transit time (PTT) or pulse arrival time (PAT) as a surrogate measure of the pulse wave velocity [8]. A linear relationship between PAT/PTT and BP can be derived from explicit analytical models [9]. However, regular calibration is required to maintain prediction accuracy. PPG-based ML is potentially a calibration-free approach [10,11] that utilizes the functional relationship between the PPG signal and blood pressure.

PPG waveform morphology reflects the elasticity and compliance of peripheral arteries, making it an ideal source for BP estimation [12]. Temporal features extracted from PPG waveforms have been found to be strong predictors of blood pressure and vascular aging [13]. Changes in the PPG waveform parallel physiological changes in BP during cold pressor stimuli and exercise [14] and infusion of a vasoconstrictive drug [15,16] as well as during sleep [17,18,19]. Existing studies using data from hospitalised patients, such as in the ICU [20,21] or under anaesthesia [22], have demonstrated a robust association between PPG and intra-arterial BP waveforms. However, these results might not be readily extrapolated to healthier persons who constitute most of the potential users of such a cuffless system. Only a few studies have evaluated healthy people. Table 1 summarizes recent studies that (1) used data from outside the ICU, (2) used the PPG signal (and features), (3) reported an error metric other than the correlation coefficient (mean absolute error (MAE) or root mean square error (RMSE)). This is important for the comparability of studies. Despite the clinical importance of nocturnal BP in CVD risk assessment, only a few studies have investigated cuffless BP prediction during sleep [23,24]. However, none of the approaches used has met the error criteria set by international guidelines from the Association for Advancement of Medical Instrumentation (AAMI)/the European Society of Hypertension (ESH)/ISO [25], which require MAE and STD values to be within 5 and 8 mmHg, respectively.

In this study, we aimed to develop a framework to estimate night-time BP using signals from the finger PPG alone. Specifically, we focused on the complex relationship between PPG waveform features and BP in healthy participants during sleep.

## 2. Materials and Methods

### 2.1. Participants

Participants were part of a sleep study that recruited nominally healthy adults aged between 21 and 70 years for a 2-night protocol in a sleep laboratory [17]. People with pre-existing sleep, neurological or psychiatric disorders, excessive daytime sleepiness (Epworth Sleepiness Scale [28] scores > 10), body mass index (BMI) > 35 kg/m^2^, and habitual sleep duration less than 5 h/night and on wake-promoting medications were excluded.

Participants visited the laboratory 3 times: First, for a daytime briefing session, where demographic and anthropometric measurements (height, weight, waist circumference, and office blood pressure measurements) were obtained. Each participant then had two overnight polysomnography (PSG) sessions in the laboratory—one session with concurrent ambulatory blood pressure monitoring (ABPM) and one session without. The order of nights with and without ABPM was randomised. Ninety participants had concurrent ABPM recording in one of their sessions.

Participants with less than 3 overnight BP measurements (*n* = 4) or no measurements in the pre-sleep baseline period (*n* = 3) and with PSG/PPG recordings of insufficient quality (*n* = 15) were excluded.

The final sample size was 68 healthy adults (53% male), with a median (IQR) age of 29 (23–46) years.

### 2.2. Laboratory Protocol

Participants arrived at the sleep laboratory approximately 2 h before their self-reported habitual bedtime. Trained research assistants set up the PSG and ABPM equipment. Participants then had approximately 15 min of pre-sleep baseline recording, during which they were asked to sit still without moving. Lights-off and lights-on times were assigned according to the participant’s habitual bedtime and wake-up time.

The Institutional Review Board of the National University of Singapore approved the study, and the protocol was in accordance with the principles in the Declaration of Helsinki. Informed written consent was obtained from all participants during the briefing session.

### 2.3. Data Acquisition

The plethysmography (PPG) signal was recorded from the fingertip concurrently with electroencephalography (EEG), electrooculography (EOG; right and left outer canthi), submental electromyography (EMG), and electrocardiography (ECG) via the SOMNOtouch system (SOMNOmedics GmbH, Randersacker, Germany) during overnight sleep. The sampling rate of PPG, EEG, and ECG was 256 Hz.

**Figure 1:**
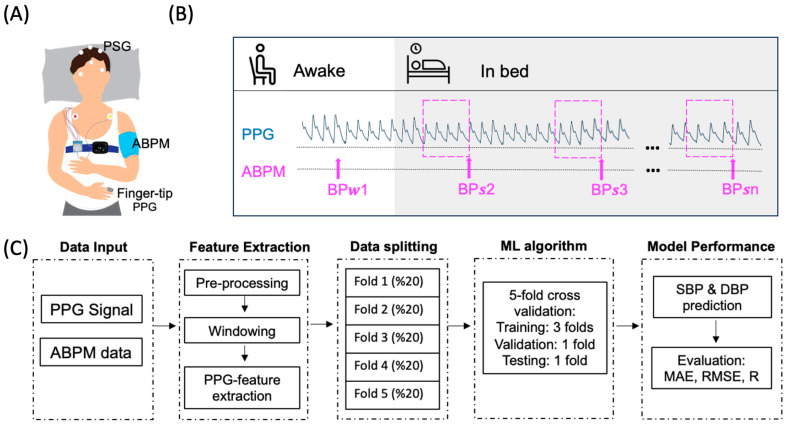
An overview of experimental protocol and analysis workflow. (**A**) Data collection setup with Polysomnography (PSG), Ambulatory Blood Pressure Monitor (ABPM), and Photoplethysmography (PPG). (**B**) PPG signal was windowed preceding ABPM measurements while in bed (BPs_n_). Awake-sitting BP measurements (BPw_n_) were excluded. (**C**) Block diagram of analysis flow.

Ambulatory blood pressure was measured using the clinically validated OnTrak 90227 monitor (Spacelabs Healthcare, Snoqualmie, WA, USA) [29] on the opposite arm of the PPG sensor. Each participant had multiple measurements during sleep, and the BP measurement interval was 30 min. Nights with less than 3 valid sleep BP measurements were excluded from the dataset. The median number of ABPM readings during sleep per participant was 15 with an IQR range of 14–16. The data acquisition protocol is summarized in Figure 1.

### 2.4. PPG Pulse Waveform Analysis and Feature Extraction

PPG waveform analyses were conducted in MATLAB version R2021b (The Math Works, Inc., Natick, MA, USA) as described in previous work [17]. The pre-processing step included band-pass filtering of the PPG signal (0.05–20 Hz via a 4th-order Chebyshev2 filter) and motion artefact reduction via amplitude thresholding. The filtered PPG was divided into 30 s windows with 20 s overlap, with pulse onsets detected within each window using the ‘qppg.m’ function from the PhysioNet Cardiovascular Signal Toolbox [30]. The following criteria were used to remove artefactual detections: (1) The onset-to-onset duration was limited to a minimum of 0.4 s and a maximum of 2 s, and (2) the maximum acceptable change in peak-to-peak duration between consecutive pulses was set to 40%. Moreover, if more than 20% of onsets (within a window) were labelled as artefacts, the entire window was discarded and not analysed further.

**Figure 2:**
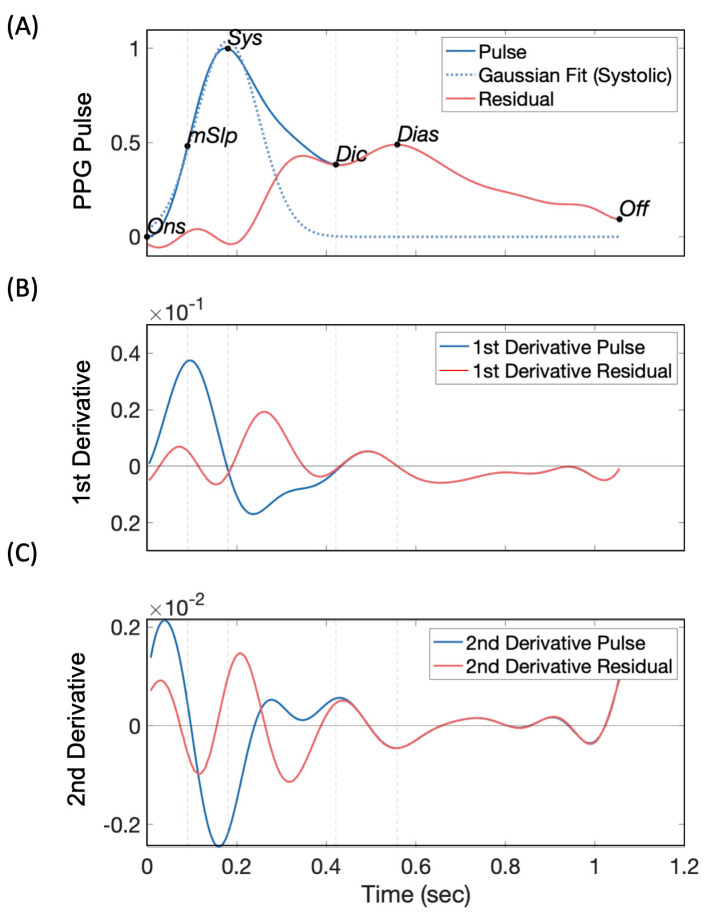
Fiducial point detection on the PPG pulse waveform using 1st and 2nd derivative signals. (**A**) PPG pulse (blue line), a Gaussian fitted to systolic phase of the pulse (dashed line) and Residual wave (red line) resulting from the subtraction of Gaussian fit from PPG pulse. (**B**) First derivatives of PPG pulse (blue line) and Residual (redline). (**C**) Second derivatives of PPG pulse (blue line) and Residual (red line). Ons: Onset, mSlp: Max Slope Point, Sys: Systolic Peak, Dic: Dicrotic Notch, Dias: Diastolic Peak, Offs: Offset.

Fiducial points of the PPG pulse (onset, offset, maximum slope point, systolic peak, diastolic peak, and the dicrotic notch) were located using a custom algorithm [17], which combined the derivatives approach [31] with Gaussian fitting [32]. First, a Gaussian (“gauss1” function from Gaussian model library) was fitted to the systolic arm of the PPG pulse (dashed blue line in Figure 2A), and its subtraction from the main pulse wave produced the residual wave (red line in Figure 2A). The max slope point was defined as the 1st peak of the 1st derivative pulse (blue line in Figure 2B), while the systolic peak was where the 1st derivative of the PPG signal crossed zero for the 1st time between max slope and offset (blue line in Figure 2B). The dicrotic notch corresponded to the largest peak on the 2nd derivative pulse wave (blue line in Figure 2C) within the window from the 2nd peak of the 1st derivative residual wave and the 3rd peak of the 1st derivative pulse. The diastolic peak was the 1st peak after dicrotic notch on the pulse wave (blue line in Figure 2A). Finally, amplitude- and duration-based PPG features were extracted for each pulse.

### 2.5. BP Prediction Model

The blood pressure prediction model was set up in Python (ver. 3.8) using PPG features in the windows preceding blood pressure measurements. A random forest regression model was trained to predict blood pressure using 75 PPG features (Supplementary Table S1) and 3 demographic features (age, sex, and BMI). Five different time window lengths (60 s, 30 s, 15 s, 7 s, and 3 s) were evaluated in terms of model performance. The random forest regression model is an ensemble of decision trees and makes predictions about the ground truth according to the vote of all of the trees [33]. This model is very capable of capturing the non-linear relationship between features and targets.

A training–validation–test procedure was used to tune the hyperparameters of the random forest regression model by using random search. More specifically, data were split into train–validation–test sets with a ratio of 3:1:1. Care was taken so that all of the trials of each participant were assigned to only 1 of the 5 partitions, which ensured that trials within a given participant were not used for both training and testing.

The random forest was trained on the training set with hyperparameters tuned on the validation set. The hyperparameters tuned were the number of trees in the forest, maximum depth of the tree, minimum number of samples required to split an internal node, minimum number of samples required to be at a leaf node, and the number of features to consider. The best hyperparameters were then used to train a final model by combining data from both training and validation sets. The prediction performance of the final model was then evaluated on the test set. At no time was the test set used to tune the hyperparameters or train the model.

To ensure stability, the above training–validation–test procedure was repeated 50 times by different random splits of the participants into training, validation, and test sets.

### 2.6. Blood Pressure Prediction Performance Evaluation

To evaluate the performance of our model, we compared the quality of predictions on the test set using mean absolute error (MAE) as the main evaluation metric. MAE measures the average absolute difference between the actual and predicted values of the target variable. Along with the MAE, the standard deviation of MAE was also reported. To assess the model’s overall performance in comparison to previous studies, we also calculated the mean root mean square error (RMSE) and mean Pearson’s correlation (R) across all individuals in the test set. Performance metrics (MAE, RMSE, and R) were first averaged across trials within each participant and then averaged across the participants. We used repeated measures correlation (to account for within-individual associations) [34] between the actual and predicted BP values. Bias was evaluated via Bland–Altman plots. Lastly, feature importance was evaluated via SHAP (Shapley additive explanations) values [35]. When comparing the two methods, the corrected resampled *t*-test was used [36,37].

## 3. Results

**Figure 3:**
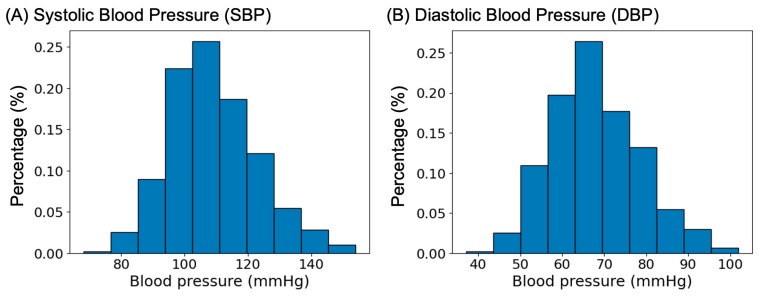
Distribution of Systolic Blood Pressure (**A**) and Diastolic Blood Pressure (**B**).

The characteristics of the sample are summarized in Table 2. The final sample consisted of 68 healthy adults whose BMI, office BP, and sleep BP (via ABPM) were in a healthy range. The median (IQR) SBP was 110 (103, 121) mmHg, and the DBP was 71 (66, 77) mmHg, while the distribution of SBP and DBP measurements is shown in Figure 3. The median number of ABPM readings during sleep per participant was 15, while the median time in bed duration was more than 7 h with a sleep efficiency of 88%.

Our model achieved the highest out-of-sample performance with a window length of 7 s out of window lengths explored (60 s, 30 s, 15 s, 7 s, and 3 s) (Table 3). The mean prediction error was 5.72 ± 4.51 mmHg (MAE ± STD) for SBP and 4.52 ± 3.60 mmHg for DBP. The same window length also resulted in the lowest RMSE for both SBP (6.47 ± 1.88 mmHg) and DBP (4.62 ± 1.17) and the highest R for SBP (0.87 ± 0.09).

On the other hand, window lengths of 3 s resulted in the worst prediction performance for all performance metrics. Mean absolute error was 6.63 ± 5.80 for SBP and 6.78 ± 5.40 for DBP, while RMSE was 6.72 ± 1.91 for SBP and 6.92 ± 1.58 for DBP. R values were also lowest with a window length of 3 s.

**Figure 4:**
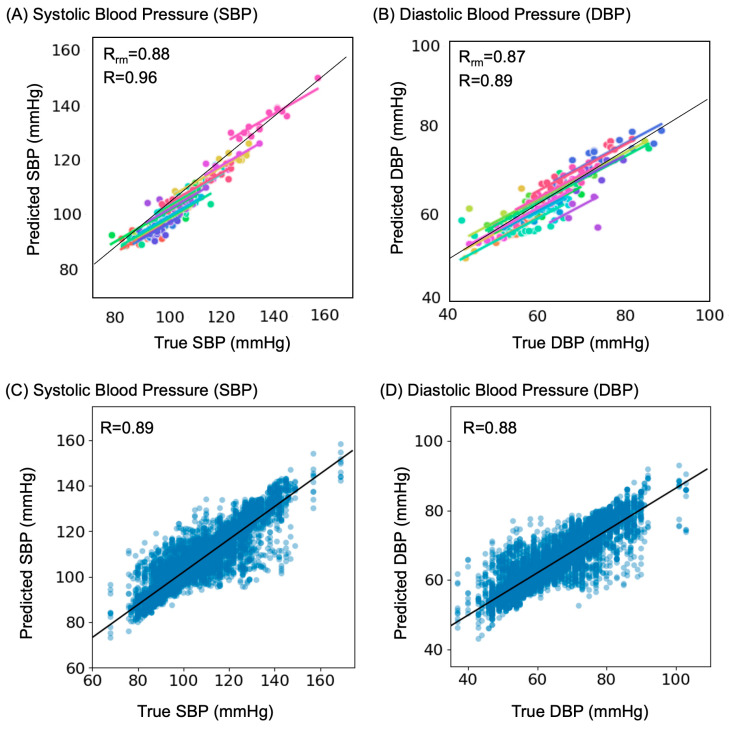
Strength of correlations between True and Predicted BP values. (**A**) Pearson correlation (R) and repeated measures correlation (R_rm_) for Systolic Blood Pressure (SBP) in a representative test set of one split (14 participants). Same colour refers to individual measurements from the same participant. (**B**) Pearson correlation (R) and repeated measures correlation (R_rm_) for Diastolic Blood Pressure (dBP) in a representative test set of one split. Same colour refers to different individual measurements from the same participant. (**C**) Pearson correlation across all sample points for SBP. (**D**) Pearson correlation across all sample points for DBP.

Next, we investigated the strength of the relationship between true and predicted BP via correlation. Figure 4, A and B exemplify results from a representative test set of one split. The Pearson correlation was 0.96 between true SBP and predicted SBP (Figure 4A) and 0.89 between true DBP and predicted DBP (Figure 4B). Similarly, the repeated measures correlation coefficient (R_rm_) was 0.88 for SBP (Figure 4A) and 0.87 for DBP (Figure 4B), indicating a strong within-individual association for this sample. Figure 4C and D depict Pearson correlations across all sample points for SBP (R = 0.89) and DBP (R = 0.88), respectively.

**Figure 5:**
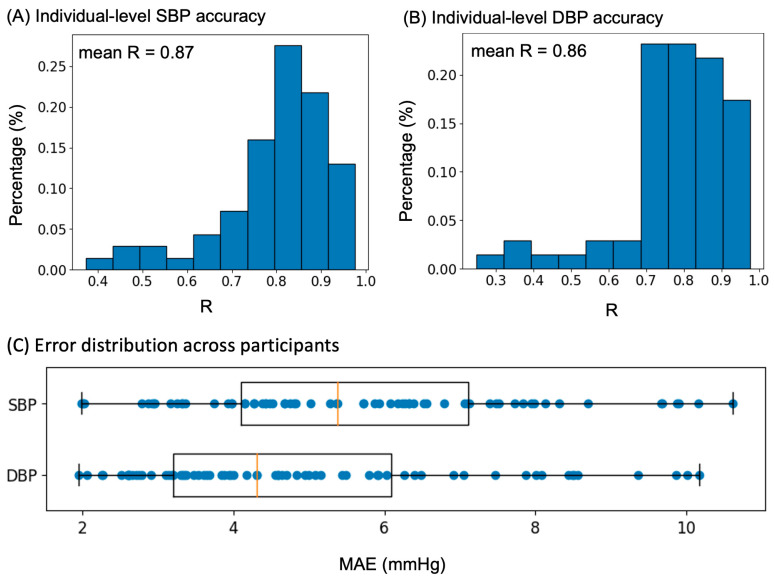
Distribution of correlation and error across participants. (**A**) Individual-level correlations across participants for Systolic Blood Pressure (SBP). First, correlation across measurement trials was calculated for each test fold of each split. Then, the average R across 10 splits for each participant was computed. (**B**) Individual-level correlations across participants for Diastolic Blood Pressure (DBP). First, correlation across trials was calculated for each test fold of each split. Then, the average R across 10 splits for each participant was computed. (**C**) Distribution of mean absolute error (MAE) across participants. The error was computed for each trial of each test subject and then averaged across all trials and splits of each participant.

We then examined how individual-level prediction accuracies were distributed across participants. Figure 5A,B display individual-level correlation coefficients for SBP and DBP, respectively. Although average R values were 0.87 for SBP and 0.86 for DBP, a few participants had poor prediction accuracy with R values below 0.7. The distribution of MAE values is shown in Figure 5C.

**Figure 6:**
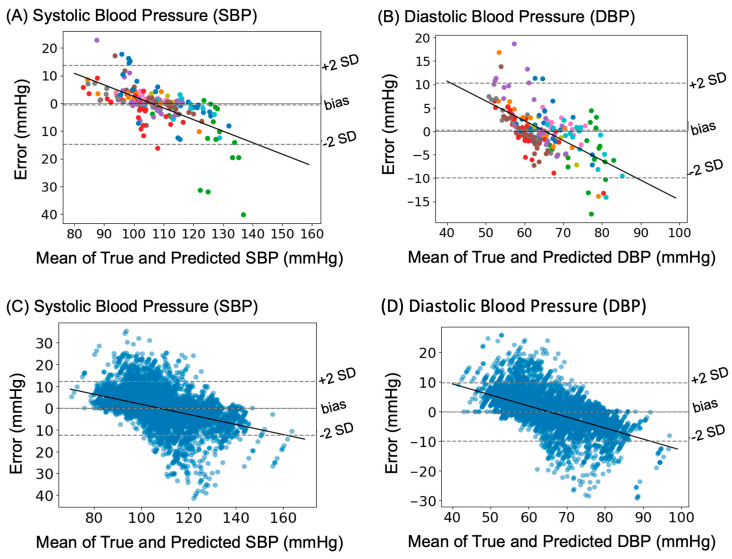
Comparison of errors between the true and predicted BP values. (**A**) Bland-Altman plot showing the agreement between True and Predicted SBP values for a representative test set of one split. Bias was −0.55 mmHg. Points of a particular colour denote different measurement trials within the same participant. (**B**) Bland-Altman plot showing the agreement between True and Predicted DBP for a representative test set of one split. Bias was 0.18 mmHg. Same colour refers to different trials within the same participant. (**C**) Bland-Altman plot across all test samples of all data splits for Systolic Blood Pressure. Bias was −0.13 mmHg. (**D**) Bland-Altman plots across all test samples of all data split for Diastolic Blood Pressure. Bias was −0.08 mmHg.

Systematic errors between true and predicted BP values are shown in Figure 6. Bland–Altman plots in Figure 6A,B exemplify the bias pattern for SBP and DBP for a representative sample (from a 1 test fold of 1 split). There was a −0.55 mmHg bias between true and predicted SBP and 0.18 mmHg bias between true and predicted DBP, with a visible proportional bias. Figure 6C,D display the error for all test samples, as an illustration of model performance. Overall, the SBP and DBP were slightly underestimated with a mean bias of −0.13 mmHg and −0.08 mmHg across all samples.

**Figure 7:**
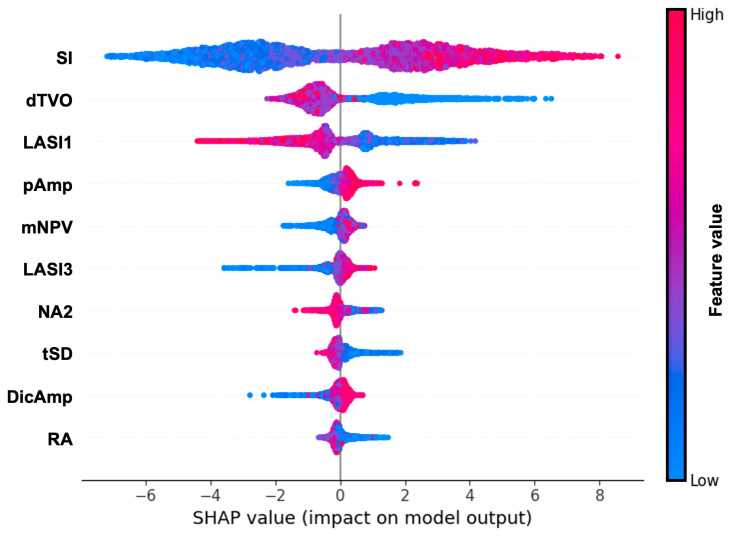
Beeswarm plot with 10 most important PPG waveform features based on SHAP values: SI: stiffness index, dTVO: minimum to offset time in first derivative, LASI1: inverse time from dicrotic notch to systolic peak, pAmp: pulse amplitude, mNPV: modified normalized pulse volume, LASI3: inverse time from diastolic peak to maximum slope point, NA2: normalized area under maximum slope point to systolic peak, tSD: descending time, DicAmp: dicrotic notch amplitude, RA: area ratios between onset to dicrotic notch and dicrotic notch to offset. Features are ordered according to their importance from top to bottom. The x-axis represents log odds. Colouring shows the direction of this association, where blue indicates lower values of a given measure and red indicates higher respective measure values.

Finally, we explored feature importance via SHAP values and listed the 10 most important PPG features according to their impact on model output in Figure 7. The SI (stiffness index), defined as the ratio between a person’s height and the distance between systolic and diastolic peaks, was the most important PPG feature followed by dTVO (global minimum to offset time in first derivative) and LASI1 (inverse time from dicrotic notch to systolic peak). When demographic variables were included in the list (Figure S1), BMI was the most important feature, followed by SI and age.

## 4. Discussion

We present a cuffless BP approach to measure nocturnal BP in a healthy sample. A random forest model was used to infer BP values using PPG waveform characteristics. The model reached an out-of-sample performance of 5.72 ± 4.51 mmHg (MAE ± STD) for SBP and 4.52 ± 3.60 mmHg for DBP using an analysis window of 7 s, demonstrating good performance in line with international guidelines [25]. Similarly, RMSE was 6.47 ± 1.88 for SBP and 4.62 ± 1.17 for DBP. Finally, we observed that the stiffness index (SI) was the most important PPG feature in terms of impact on model output, highlighting the complex and dynamic relationship between vascular stiffness, blood pressure, and sleep.

### 4.1. Nocturnal BP Prediction Using PPG Waveform

PPG-based BP prediction via ML is a calibration-free approach that can exploit a single or multiple channels of PPG as input. The PPG pulse contour might vary due to functional (e.g., autonomic nervous system activity during sleep [17,18,19] and physical activity [14]) or structural changes (e.g., ageing [38,39] and/or disease [40]). The temporal features extracted from the PPG pulse have been shown to be strong predictors of blood pressure and vascular aging [13,16]. For example, the time interval and amplitude ratio between the first and second peaks of PPG are related to arterial properties and inversely correlated with age [15,38,39], while pulse amplitude may reflect vasoconstriction due to increased sympathetic activity [19,41]. Although there is no consensus on which PPG features are most indicative of arterial function, several studies employed BP prediction models using PPG features in healthy people. Most models focused on predicting daytime BP (Table 1). These studies differed in sample characteristics such as age (mostly middle age and above) and health status (healthy [26,27] or mixed with patients [7,8]) and the set of features used in models (PPG features alone [6,7,26] or in combination with PTT [8,27]). Although daytime BP prediction models have achieved good prediction performance [8,27], an important limitation is that the user is required to deliberately avoid movement during recording.

Sleep provides a unique opportunity to monitor BP levels with reduced levels of movement artefacts. Despite the clinical importance of nocturnal BP in CVD risk assessment, there are only a few studies predicting night-time BP. A PAT-based model was employed to predict 24 h BP in 10 healthy adults, and the performance during sleep was suboptimal with an RMSE of 8.2 mmHg for SBP and 6.7 mmHg for DBP [24]. More recently, 24 h BP trends and BP dipping were predicted using features of the wrist PPG signal in healthy participants [23]. The use of a long- and short-term memory network (LSTM) model yielded an RMSE of 8.22 ± 2.11 mmHg for relative SBP and 6.76 ± 1.48 mmHg for relative DBP, with correlation coefficients of 0.69 and 0.76 for SBP and DBP, respectively. In the same study, a random forest model was also evaluated but had poorer performance than the LSTM model. Although direct comparison was not possible due to differences in error metrics (MAE vs. RMSE) and prediction target (absolute BP vs. relative BP), both studies highlighted the potential of night-time BP prediction using PPG.

### 4.2. Pulse Waveform Modulation during Sleep

Compared to stable daytime measurements or ICU recordings, PPG signals during sleep fluctuate more widely. However, they are less susceptible to movement artefacts. This makes sleep an ideal state for the detection of nocturnal hypertension that could otherwise be masked during the day. During sleep, several physiological changes that affect the shape of the PPG waveform occur. For example, a shift towards sympathetic activity increases vascular tone and decreases blood flow, resulting in a lower PPG pulse amplitude [42,43]. Decreases in pulse wave amplitude have been associated with obstructive respiratory events and cortical arousals as a marker of an increased sympathetic drive [41,43]. Similarly, the time difference between peaks of PPG pulse, which is an indicator of arterial stiffness, changes according to sleep stage, lengthening during deeper stages of sleep [17,18]. Thus, the PPG signal carries useful information about cardiac contractility, vascular elasticity, and autonomic nervous system activity, three key determinants of blood pressure. Our study also showed that for the time difference between peaks and pulse amplitude, the features that had the greatest impact on model output (Figure 6) were indeed waveform characteristics modulated by sleep stage [17].

### 4.3. Clinical Importance of Nocturnal BP Measurement

In population studies, both daytime SBP and nocturnal BP measures have been shown to be independent risk factors for cardiovascular and cerebrovascular disease and mortality [4,44]. While wearable cuffless BP devices have been validated for daytime usage [45,46], their performance during sleep remains unclear. Traditionally, the detection and monitoring of nocturnal hypertension requires a cuff-based ABPM to be worn during sleep [47]. This can be uncomfortable and might disrupt sleep. When we looked at the effect of ABPM on sleep, we also saw that there was a slight reduction in sleep quality and amount of deep sleep in sessions with ABPM compared to sessions without ABPM (Table S2). Due to the high cost, the technology is also limited to clinical use and cannot be deployed on a large scale as a longitudinal monitoring system. Therefore, the emergence of a cuffless, non-invasive BP measurement device using physiological signals offers a compelling alternative, especially considering the widespread use and accessibility of modern sleep trackers equipped with advanced PPG sensors.

Despite the clinical importance, only a limited number of studies have attempted to predict night-time BP via a cuffless approach. Likewise, a significant number of PPG-based algorithms were developed using data from intensive care patients (such as the MIMIC dataset), and they are often difficult to generalize to healthy populations. Here, we proposed an algorithm to predict nocturnal BP in healthy people, which is generally overlooked in prediction studies. Starting to monitor nocturnal BP in good health—before the onset of hypertension—is important to establish one’s ‘baseline and patterns’ that could help in the formulation of strategies to reduce the impact of future hypertension in susceptible individuals.

### 4.4. Limitations

Our model required only a single-channel PPG signal and a signal window length of 7 s to achieve a high performance close to the error limits set by international guidelines [25]. On the other hand, because our sample comprised young and healthy people, the range of BP values assessed was relatively restricted. The generalization of our model to older persons, those with hypertension, and those with peripheral vascular disease remains to be shown. The proportional bias towards higher blood pressure estimates when true values are below the sample mean and lower blood pressure when true values are higher than the sample mean may make the method suited mainly for studying the nocturnal physiology of healthier normotensive persons.

## 5. Conclusions

A machine-learning-based approach based on PPG waveform features during sleep can provide a scalable, convenient, economical, and non-invasive means to continuously monitor blood pressure. With the widespread use of sleep trackers with inbuilt PPG sensors, this may prove to be an approach that allows for large-scale evaluation of nocturnal BP fluctuations in currently normotensive persons and possibly a tool for identifying persons at risk for cardiovascular or cerebrovascular disease.

## Figures and Tables

**Table 1:** Studies that used ML models to predict BP using PPG features. The list is not exhaustive. A study was included if (1) data were from community-dwelling adults, (2) PPG waveform features were used, and (3) results were reported as MAE or RMSE. Studies that used deep neural networks and raw PPG signals as input were excluded. N.R: Not Reported.

Study	Target BP	Reference Measure	PPG Location	Sample, Size, Age	Input Format	Method	Error (mmHg)	
							SBP	DBP
[6]	Daytime	Point BP	Finger	Health N.R., N = 205, 39 ± 15 yo	PPG Features	Lasso	MAE: 6.9	MAE: 5
[26]	Daytime	Point BP	Forearm	Healthy, N = 40, 35 ± 15 yo	PPG Features	Ensemble SVR	MAE: 7.26	MAE: 5.01
[7]	Daytime	Point BP	Finger	Outpatients, N = 219, 57 ± 15 yo	PPG Features	GPR	MAE: 3.02 RMSE: 6.74	MAE: 1.74 RMSE: 3.59
[8]	Daytime	Finapress*, Point BP	Finger	Mixed, N = 62, 59 ± 10 yo	PPG Features + PTT	GPR	MAE: 4.8	MAE: 3.4
[27]	Daytime	Finapress*, Point BP	Finger	Healthy, N = 84, Age N.R.	PPG Features + PTT	Deep RNN	RMSE: 3.9	RMSE: 2.6
[23]	24 h trend	ABPM	Wrist	Healthy, N = 106, 36.6 ± 11.7 yo	PPG Features	LSTM	RMSE: 8.2	RMSE: 6.5
This work	Night-time	ABPM	Finger	Healthy, N = 68, 29 (23–46) yo	PPG Features	Random forest	MAE: 5.7 RMSE: 6.5	MAE: 4.5 RMSE: 4.6

* Continuous blood pressure monitor.

**Table 2:** Summary of participant characteristics. SBP: Systolic blood pressure, DBP: Diastolic Blood Pressure, ABPM: Ambulatory Blood Pressure.

Sample Summary	N = 68 ^1^
Age	29 (23, 46)
Gender	
Female	32 (47%)
Male	36 (53%)
BMI (kg/m^2^)	22.8 (21.1, 24.3)
Office SBP (mmHg)	110 (103, 121)
Office DBP (mmHg)	71 (66, 77)
**ABPM Summary**	
Sleep SBP (mmHg)	104 (99, 114)
Sleep DPB (mmHg)	64 (60, 69)
BP readings per participant (*n*)	15 (14, 16)
**Sleep Summary**	
Time in Bed (min)	463 (420, 490)
Sleep Efficiency (%)	88 (79, 92)

^1^ Median (IQR); *n* (%).

**Table 3:** Prediction performance for Systolic Blood Pressure and Diastolic Blood Pressure using different window lengths. Each performance metric was first averaged across all measurement trials within participants and then averaged across all participants. MAE: Mean Absolute Error, RMSE: Root Mean Square Error, R: Pearson Correlation, SD: Standard Deviation.

Window Length (s)	Systolic Blood Pressure			Diastolic Blood Pressure		
	MAE (SD)	RMSE (SD)	R (SD)	MAE (SD)	RMSE (SD)	R (SD)
1 min	6.38	6.51	0.866	4.92	5.14	0.864
	(−4.61)	(−1.87)	(−0.08)	(−3.63)	(−1.12)	(−0.06)
30 s	6.09	6.53	0.868	4.81	4.92	0.869
	(−4.46)	(−1.87)	(−0.09)	(−3.52)	(−1.14)	(−0.08)
15 s	5.9	6.48	0.869	4.64	4.67	0.871
	(−4.61)	(−1.76)	(−0.09)	(−3.54)	(−1.11)	(−0.08)
7 s	5.72	6.47	0.87	4.52	4.62	0.868
	(−4.51)	(−1.88)	(−0.09)	(−3.6)	(−1.17)	(−0.08)
3 s	6.63	6.72	0.865	6.78	6.92	0.857
	(−5.8)	(−1.91)	(−0.08)	(−4.5)	(−1.58)	(−0.08)

## Data Availability

The data presented in this study are available upon reasonable request from the first and the corresponding authors.

## Supplementary Materials

The following supporting information can be downloaded at: https://www.mdpi.com/article/10.3390/s23187931/s1, Table S1: List of PPG waveform features used in models, Figure S1: Beeswarm plot with most important features (including demographic features) based on SHAP values.
