# Supplementary material for: Nocturnal Blood Pressure Estimation from Sleep Plethysmography Using Machine Learning
Source: Sensors (Basel). 2023 Sep 16;23(18):7931. doi: 10.3390/s23187931 (PMC10537552; doi:10.3390/s23187931)
Supplement: Supplementary file 1 [file sensors-23-07931-s001.zip › sensors-2605200-supplementary.pdf]

sensors

MDPI

Supplementary Material

Article

Nocturnal Blood Pressure Estimation from Sleep Plethysmography Using Machine Learning

Gizem Yilmaz [1,‡], Xingyu Lyu [1,2,‡], Ju Lynn Ong [1], Lieng Hsi Ling [3,4], Thomas Penzel [5], B. T. Thomas Yeo [1,2,6,7,8,9,*] and Michael W. L. Chee [1,*]

1 Centre for Sleep and Cognition, Yong Loo Lin School of Medicine, National University of Singapore, Singapore; gizem.yilmaz@nus.edu.sg (G.Y.); lyux96@nus.edu.sg (X.L.); julynn.ong@nus.edu.sg (J.L.O.)
2 Centre for Translational Magnetic Resonance Research (TMR), Yong Loo Lin School of Medicine, National University of Singapore, Singapore
3 National University Heart Centre, Singapore; mdcllh@nus.edu.sg (L.H.L.)
4 Department of Medicine, Yong Loo Lin School of Medicine, National University of Singapore, Singapore
5 Interdisciplinary Center of Sleep Medicine, Charité—Universitätsmedizin Berlin, Berlin, Germany; thomas.penzel@charite.de (T.P.)
6 Department of Electrical and Computer Engineering, National University of Singapore, Singapore
7 N.1 Institute for Health and Institute for Digital Medicine (WisDM), National University of Singapore, Singapore
8 Integrative Sciences and Engineering Programme (ISEP), National University of Singapore, Singapore
9 Martinos Center for Biomedical Imaging, Massachusetts General Hospital, Charlestown, MA, USA
* Correspondence: thomas.yeo@nus.edu.sg (B.T.T.Y.); michael.chee@nus.edu.sg (M.W.L.C.)
‡ These authors contributed equally.

Table S1. The list of PPG waveform features used in prediction models

**PPG features and description**
A1: Area from onset to max slope point
A2: Area from max slope point to systolic peak
A3: Area from systolic peak to dicrotic_notch
A4: Area from dicrotic notch to diastolic peak
A5: Area from onset to offset
S1: ratio between A1 and A2
S2: ratio between A1 and A3
S3: ratio between A1 and A4
S4: ratio between A1 and A5
S5: ratio between A2 and A3
S6: ratio between A2 and A4
S7: ratio between A2 and A5
S8: ratio between A3 and A4
S9: ratio between A3 and A5
S10: ratio between A4 and A5
S11: ratio between A1-4 and A5
AA: Ascending area
Daa: Ascending area for first derivative
sdAA: Ascending area for second derivative
DA: Descending area
dDA: Descending area for first derivative
sdDA: Descending area for second derivative
RA: ratio between (A1+A2+A3) and A4
dRA: ratio between dAA and dDA
sdRA: ratio between sdAA and sdDA
NA1: Normalized-area from onset to max slope point
NA2: Normalized-area from max slope point to systolic peak
NA3: Normalized-area from systolic peak to dicrotic_notch
NA4: Normalized-area from dicrotic notch to diastolic peak
NA5: Normalized-area from onset to offset
NS1: ratio between NA1 and NA2
NS2: ratio between NA1 and NA3
NS3: ratio between NA1 and NA4
NS4: ratio between NA1 and NA5
NS5: ratio between NA2 and NA3
NS6: ratio between NA2 and NA4
NS7: ratio between NA2 and NA5
NS8: ratio between NA3 and NA4
NS9: ratio between NA3 and NA5
NS10: ratio between NA4 and NA5
NS11: ratio between NA1-4 and NA5
PI: pulse intensity
dPI: pulse intensity for first derivative
sdPI: pulse intensity for second derivative
dicAmp: dicrotic notch intensity
AID: onset intensity
DID: offset intensity
pAmp: pulse average intensity
RI1: ratio between intensity of max slope point and PI

RI2: ratio between NI and PI
RI3: ratio between intensity of diastolic peak and PI;
lnRI: log(RI1);
lnRI2: log(RI2);
lnRI3: log(RI3);
AI2: ratio between (PI-NI) and PI;
ppg_dc: Mean PPG signal intensity
ppg_ac: Intensity between systolic peak and onset
mNPV1: ratio between ppg_ac and ppg_dc
mNPV2: ratio between ppg_ac and (ppg_ac+ppg_dc);
CC: Cardiac circle time
tSA: Ascending time
tSD: Descending time
dDT: Descending time for first derivative
sdDT: Descending time for second derivative
dTVO: Min of der1 to offset time
sdTVO: Min of der2 to offset time
SD: Onset to dicrotic notch time
DD: Dicrotic notch to offset time
RSD: ratio between SD and DD;
RSC: ratio between SD and CC;
RDC: ratio between DD and CC
LASI1: inverse of time between dicrotic notch and systolic peak
LASI2: inverse of time between diastolic peak-systolic peak
LASI3: inverse of time between diastolic peak-max slope point
LASI4: inverse of time between systolic peak-max slope point
HR: heart rate
lnHR: log of heart rate
expHR: exp of heart rate
SI: stiffness index
mNPV: modified normalized pulse volume

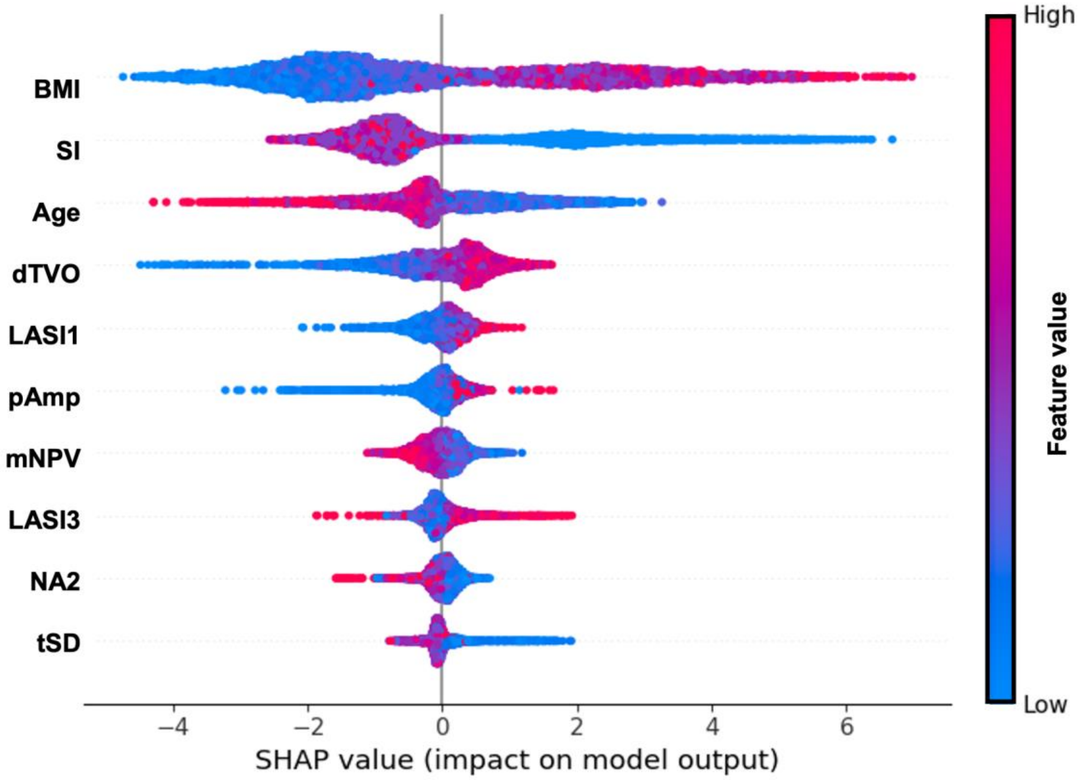

Figure S1. Beeswarm plot with 10 most important features including demographic feaures based on SHAP values: BMI: Body mass index, SI: stiffness index, Age, dTVO: minimum to offset time in first derivative, LASI1: inverse time from dicrotic notch to systolic peak, pAmp: pulse amplitude, mNPV: modified normalized pulse volume, LASI3: inverse time from diastolic peak to maximum slope point, NA2: normalized area under maximum slope point to systolic peak, tSD: descending time. Features are ordered according to their importance, from top to bottom. The x-axis represents log odds. Colouring shows the direction of this association, where blue indicates lower values of a given measure and red indicates higher respective measure values.

Table S2. Paired comparison of sleep variables on nights without ABPM (ABPMoff) and with ABPM (ABPMon) for participants who had data in both nights (N=45).

**Variable**	**ABPMoff**, N = 45	**ABPMon**, N = 45	**p-value**[1]
**TIB (min)**			0.5
Mean (SD)	456.6 (49.0)	459.0 (50.0)	
Range	330.5, 565.5	343.5, 562.5	
**TST (min)**			0.13
Mean (SD)	404.0 (48.5)	391.9 (56.1)	
Range	306.5, 517.5	262.5, 504.0	
**SOL (min)**			0.5
Mean (SD)	17.8 (12.0)	19.1 (15.7)	
Range	2.5, 52.5	0.0, 73.5	
**WASO (min)**			**0.043**

**Variable**	**ABPMoff**, N = 45	**ABPMon**, N = 45	**p-value**[1]
Mean (SD)	30.2 (23.9)	42.3 (34.1)	
Range	2.5, 102.5	3.5, 134.5	
**N3 (min)**			**0.007**
Mean (SD)	76.5 (28.2)	65.0 (31.2)	
Range	12.0, 153.5	8.5, 131.5	
**N2 (min)**			0.7
Mean (SD)	228.2 (36.9)	230.1 (35.2)	
Range	149.5, 309.5	142.0, 308.5	
**N1 (min)**			**0.013**
Mean (SD)	13.5 (7.1)	17.8 (13.0)	
Range	1.5, 32.5	3.5, 69.5	
**REM (min)**			0.15
Mean (SD)	85.9 (35.2)	79.0 (28.3)	
Range	2.0, 153.5	18.5, 145.5	
**NREM (min)**			0.4
Mean (SD)	318.1 (34.0)	312.9 (39.8)	
Range	222.5, 388.5	233.0, 389.5	
**SE (%)**			**0.036**
Mean (SD)	88.7 (6.9)	85.5 (9.4)	
Range	70.4, 97.5	61.4, 99.1	

[1]Paired t-test
